# Interspecies ecological competition rejuvenates decayed *Geobacter* electroactive biofilm

**DOI:** 10.1093/ismejo/wrae118

**Published:** 2024-06-25

**Authors:** Yin Ye, Lu Zhang, Xiaohui Hong, Man Chen, Xing Liu, Shungui Zhou

**Affiliations:** Fujian Provincial Key Laboratory of Soil Environmental Health and Regulation, College of Resources and Environment, Fujian Agriculture and Forestry University, Fuzhou 350002, China; Fujian Provincial Key Laboratory of Soil Environmental Health and Regulation, College of Resources and Environment, Fujian Agriculture and Forestry University, Fuzhou 350002, China; Fujian Provincial Key Laboratory of Soil Environmental Health and Regulation, College of Resources and Environment, Fujian Agriculture and Forestry University, Fuzhou 350002, China; Fujian Provincial Key Laboratory of Soil Environmental Health and Regulation, College of Resources and Environment, Fujian Agriculture and Forestry University, Fuzhou 350002, China; Fujian Provincial Key Laboratory of Soil Environmental Health and Regulation, College of Resources and Environment, Fujian Agriculture and Forestry University, Fuzhou 350002, China; Fujian Provincial Key Laboratory of Soil Environmental Health and Regulation, College of Resources and Environment, Fujian Agriculture and Forestry University, Fuzhou 350002, China

**Keywords:** Geobacter species, bioelectrochemical system, electroactive biofilm, prophage induction, ecological competition, biofilm decay

## Abstract

Bioelectrochemical systems (BESs) exploit electroactive biofilms (EABs) for promising applications in biosensing, wastewater treatment, energy production, and chemical biosynthesis. However, during the operation of BESs, EABs inevitably decay. Seeking approaches to rejuvenate decayed EABs is critical for the sustainability and practical application of BESs. Prophage induction has been recognized as the primary reason for EAB decay. Herein, we report that introducing a competitive species of *Geobacter uraniireducens* suspended prophage induction in *Geobacter sulfurreducens* and thereby rejuvenated the decayed *G. sulfurreducens* EAB. The transcriptomic profile of *G. sulfurreducens* demonstrated that the addition of *G. uraniireducens* significantly affected the expression of metabolism- and stress response system-related genes and in particular suppressed the induction of phage-related genes. Mechanistic analyses revealed that interspecies ecological competition exerted by *G. uraniireducens* suppressed prophage induction. Our findings not only reveal a novel strategy to rejuvenate decayed EABs, which is significant for the sustainability of BESs, but also provide new knowledge for understanding phage–host interactions from an ecological perspective, with implications for developing therapies to defend against phage attack.

## Graphical Abstract

**Graphical Abstract:**
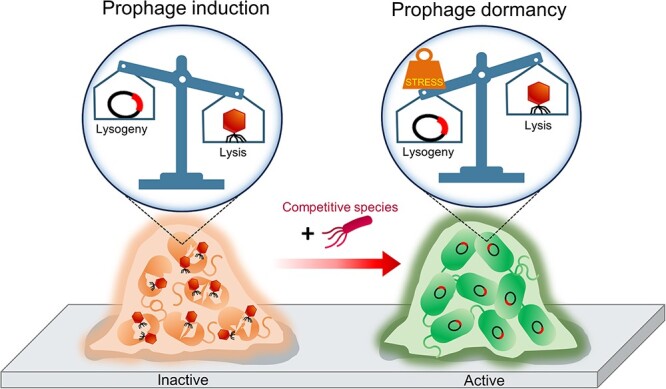

## Introduction

Bioelectrochemical systems (BESs) utilize the ability of electroactive bacteria to synchronize intracellular biocatalytic redox reactions with extracellular electron exchange with an electrode and therefore hold great promise in wastewater treatment; green energy, such as electricity and hydrogen production; value-added chemical biosynthesis; and biosensing [1–3]. In these BESs, electroactive bacteria usually attach to electrodes to form electroactive biofilms (EABs) [4]. Moreover, various methods have been developed to induce the formation of EABs [5, 6]. This is because it is generally recognized that forming EABs is necessary to achieve efficient electron exchange between electroactive bacteria and electrodes. Furthermore, sustaining the activity of EABs determines the high performance and work efficiency of BESs [7]. However, in most cases, EABs gradually decay with the operation of BESs and the performance of the BESs declines [8, 9]. Unfortunately, to date, no effective methods have been developed to rejuvenate decayed EABs, aside from the suggestion to regularly erase the decayed EABs from the electrodes to induce EAB regrowth [9, 10].

*Geobacter sulfurreducens* is the type of electroactive bacterium that generates the highest current in BESs and widely exists in various EABs [11, 12]. It can reduce the anode directly by expressing abundant cytochromes and nanowires and thereafter form a thick biofilm of tens of micrometers on the anode [13]. However, the *G. sulfurreducens* EAB does not always metabolize actively as a whole. During the operation of the BES, *G. sulfurreducens* EAB decays due to the accumulation of dead cells and, meanwhile, the current generation declines [14–16]. Nutrient substance consumption and toxic metabolite accumulation have been suggested to be the reasons for the decay of *G. sulfurreducens* EAB [17–20]. In addition, the attenuation of the redox gradient across *G. sulfurreducens* EAB during EAB development has also been thought to contribute to the decay [15]. However, a recent study indicated that all of these factors might trigger the lysogenic to lytic transition of prophages in *G. sulfurreducens* cells, and phage attack is the primary and internal reason for *G. sulfurreducens* EAB decay [21].

Phages have both lysogenic and lytic life stages [22]. The lysogenic stage can have neutral or beneficial effects on the host cell, but the lytic stage only has detrimental effects [23–26]. Thus, blocking the lysogenic to lytic transition of prophages will prevent cell lysis triggered by phages. Primary efforts have been made to interfere with this lytic transition. For example, the genetically activating and controlling repressor protein CI or other cleavable repressors and the holin protein of the host cell impedes prophages from switching to the lytic cycle [27]; addition of protease inhibitors such as antipain inhibits prophage induction in microbes by blocking proteolytic inactivation of repressors [28]; increasing the amount of signal peptides, such as hexapeptide moieties, promotes the ability of phages to commit to the lysogenic cycle [29]. However, these methods are only exclusive to some microbes and, to an extent, harm microbes in other physiological aspects.

The status of the host cell could also affect the prophage transition. For instance, when cells grow under optimal conditions, prophages can voluntarily transit to lytic cycles [30–32]; on the contrary, when hosts are under stress conditions, such as elevated or decreased temperatures (or pH) [33], overwhelming osmotic pressure [34], or nutrient deficiency [35], many prophages will predominantly stay in lysogenization. In this study, we found that introducing ecological competition via the addition of a competitive species, *Geobacter uraniireducens*, suppressed prophage induction in *G. sulfurreducens* and subsequently rejuvenated the decayed *G. sulfurreducens* EAB. Our findings provide the first strategy to revitalize a decayed EAB and raise new knowledge for understanding the phage life cycle and phage–host interactions.

## Materials and methods

### Bacterial strains and culture conditions

The *G. sulfurreducens* strain PCA (ATCC 51573), *G. uraniireducens* strain Rf4 (ATCC BAA-1134), *Geobacter bemidiensis* strain Bem (ATCC BAA-1014), and *Escherichia coli* DH5α were acquired from our laboratory culture collection. *Geobacter sulfurreducens*, *G. uraniireducens*, and *G. bemidiensis* were routinely cultured at 30°C under strict anoxic conditions (80:20 N_2_/CO_2_) in nutrient broth (NB) media supplemented with 15 mM sodium acetate as the electron donor and 40 mM fumarate as the electron acceptor, as previously reported [36]. *Escherichia coli* was routinely cultured at 37°C in Luria–Bertani medium. Cell growth was monitored by measuring optical density of the culture at 600 nm.

### BES construction and electrochemical measurement

A three-electrode system consisting of a dual-chambered H-shaped bioelectrochemical reactor was constructed with the anode and the cathode chambers separated by a proton exchange membrane as previously described [37]. In both chambers, polished graphite carbon plates with dimensions of 30 × 20 × 3 mm were used as electrodes, 100 mL of an anaerobic freshwater medium (FWNN) was used as the electrolyte, and 15 mM acetate was supplied in the anode chamber as the sole electron donor [38]. The inoculum was prepared from a total of 10 mL of mid-exponential *G. sulfurreducens* culture grown in NBAF medium that was centrifuged at 8000 × g for 8 min and washed twice with FWNN. A constant voltage of +0.3 V (vs. Hg/HgCl sat. KCl) was applied to the working electrode, and the current was monitored continuously with a 1000C electrochemical workstation (CH Instrument Inc., China). The system was operated in batch mode. When the current decreased to less than 10^−3^ mA, the electrolyte was replaced with fresh medium supplemented with 15 mM acetate.

Cyclic voltammetry was conducted with a CHI760E electrochemical workstation (CH Instrument Inc., Shanghai, China) by scanning the potential from −0.6 to 0.3 V with a gradient scanning rate. Electrochemical impedance spectroscopy was performed with a CHI760E electrochemical workstation at an open-circuit voltage with a sinusoidal perturbation amplitude of 5 mV over a frequency range from 10 000 to 0.1 Hz to analyze the internal impedances of the anode biofilms. All tests were performed under anoxic conditions (80:20 N_2_/CO_2_) at 30°C.

### Biofilm viability analysis

The anodes were removed from the bioelectrochemical reactors, rinsed with 0.9% NaCl to wash out the floating cells, and then stained with Live/Dead stain (Live/Dead BacLight bacterial viability kit; Thermo Fisher Scientific, USA) for 15 min in the dark following the manufacturer’s instructions. Thereafter, the stained biofilm was visualized by a confocal laser scanning microscope (Carl Zeiss, Germany) equipped with a 20× objective lens at excitation wavelengths of 488 nm (SYTO 9, live cells) and 543 nm (propidium iodide, dead cells). The specific viability of each biofilm layer was determined by calculating the ratio of viable/total cells per area (obj/total) with Image-Pro Plus software as previously described [39]. The total viability of an EAB was calculated as the average viability of each biofilm layer.

### Bacterial quantification

The relative abundances of *G. sulfurreducens* and *G. uraniireducens* were quantified by quantitative PCR (qPCR). Cells were collected, and whole-genome DNA was extracted using the FastDNA Spin Kit (MP Biomedicals, Santa Ana, USA). Species-specific primers were designed for *G. sulfurreducens* and *G. uraniireducens* (Table S1). The DNA fragments amplified with the species-specific primers were TA-cloned and inserted into the T vector pMD19-T (Takara) and then verified by sequencing (Sango Biotech, Shanghai, China). The resulting plasmids were utilized for the preparation of the standard curves. The amounts of each species were determined by calculating against the standard curves.

### Measurement of niche and relative fitness differences

Mutual invasion experiments were conducted to determine the interspecies relationship of coexistence or competitive exclusion between *G sulfurreducens* and the other three species of *G. uraniireducens*, *G. bemidiensis*, and *E. coli*, respectively, as previously described [40, 41]. Specifically, one species acted as an invasive species was inoculated into a steady-state culture of the other species and was inoculated alone into a fresh culture medium, separately, and the growth of the invasive species was measured by qPCR. Thereafter, the growth rates of the invasive species in each situation (*μ*_invading_ and *μ*_alone_) over a 3-day period were calculated according to the following equation:


(1) \begin{equation*} \mu =\frac{1}{T}\cdot \ln \frac{D_T}{D_0}, \end{equation*}

where *D_0_* and *D_T_* are the cell densities of invasive species at Day 1 and 3, respectively, and T = 3. The sensitivity (*S*) of the invasive species to competition was calculated as follows:


(2) \begin{equation*} S=\frac{\mu_{\mathrm{alone}}-{\mu}_{\mathrm{invading}}}{\mu_{\mathrm{alone}}}\ . \end{equation*}

The niche difference (ND) and relative fitness difference (RFD) were calculated as follows:


(3) \begin{equation*} \mathrm{ND}=1-\sqrt{S_a{S}_b} \end{equation*}

(4) \begin{equation*} \mathrm{RFD}=\sqrt{S_a/{S}_b}, \end{equation*}

where *S_a_* and *S_b_* represent the sensitivity to competition of species *a* and *b*, respectively, when they are invaded mutually.

### Flow cytometric enumeration of phage particles

The anolyte was harvested and centrifuged to remove cells. The supernatant was further filtered through a 0.22-μm filter membrane (Merck, Darmstadt, Germany). To remove extracellular vesicles, one volume of chloroform was added to the filtrate. The mixture was incubated at room temperature for 10 min and then centrifuged at 10 000 × g at 4°C for 5 min. Further treatment of the sample for flow cytometric enumeration of phage particles was performed as described in a previous study [42]. Briefly, the supernatant was collected and further fixed with 0.5% v/v glutaraldehyde. After diluting 10^3^-fold with 0.1 M TE buffer (pH 8), the sample was stained with SYBR Green I (Sigma–Aldrich, St. Louis, USA) at 80°C for 10 min in the dark and then cooled to room temperature prior to analysis via flow cytometry (Thermo Fisher Scientific, Waltham, USA). Phage particles were enumerated using a mixed function that included the cytometer flow rate, sample volume, sample dilution, and number of positive events within the gated region of interest.

### Transcriptomic analysis

Anode biofilms were collected by scraping, and total RNA was extracted using the HiPure Universal RNA Mini Kit (Magen Biotechnology Co., Ltd., Guangzhou, China) according to the manufacturer’s instructions. Whole mRNA-seq libraries were prepared by Guangdong Magigene Biotechnology Co., Ltd. (China) using the NEBNext Ultr Directional RNA Library Prep Kit and sequenced on a NovaSeq 6000 System (Illumina). The raw data were processed by Trimmomatic (v.0.36) to obtain clean reads, which were then mapped to the NCBI Rfam database to remove rRNA sequences via Bowtie2 (v2.33). The remaining mRNA sequences were further mapped against the genome of *G. sulfurreducens* (NC_002939.5) by HISAT2 (2.1.0). Mapped reads were normalized to fragments per kilobase of exon per million to ensure that the expression levels of the genes were comparable among the groups with different treatments.

### Quantification of phage-related gene expression

Total RNA was treated with the Evo M-MLV RT Mix Kit with gDNA Clean for qPCR Ver.2 (Accurate Biology, Changsha, China) to remove genomic DNA, and reverse transcription was subsequently performed. Thereafter, qPCR was performed to quantify the expression of the *gp17* (GSU2178) and *gp19* (GSU2163) genes from prophage region 4 with gene-specific primers using the LightCycler 480 System (Roche Applied Science, Penzberg, Germany). The housekeeping gene *rpoD* was selected as the internal reference gene for *G. sulfurreducens*. All primers used are listed in Table S1.

## Results

### The addition of *G. uraniireducens* rejuvenates the decayed *G. sulfurreducens* EAB

**Figure 1:**
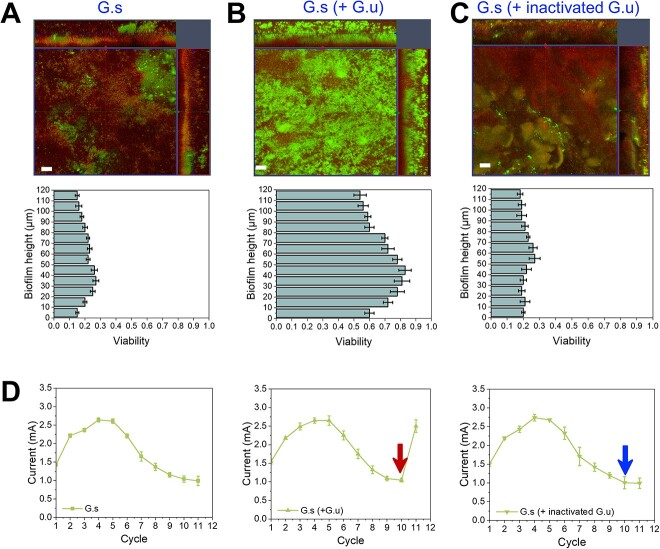
The metabolic activity and current generation of *G. sulfurreducens* biofilms; confocal laser scanning microscopy images and depth-related viability profiles of *G. sulfurreducens* biofilms before (A) and after treatment with active *G. uraniireducens* (B) or cell-free lysate of *G. uraniireducens* (inactive) (C); 5 mL of *G. uraniireducens* culture with an OD600 of 0.35 was added. *Geobacter sulfurreducens* biofilms growing in the 10th batch were tested, and biofilms from the 11th batch were examined when a peak current was generated, and biofilms were stained with live/dead stain; live cells were imaged as green, dead cells were imaged as red, and representative images were selected from three independent experiments, and the scale bars represent 30 μm, and for the viability calculation, at least five different sites of the anode biofilm were evaluated; (D) peak current profiles of *G. sulfurreducens* during 11 batches of operation; the arrows indicate the addition of *G. uraniireducens* (+ G.u) and the addition of cell-free lysate of *G. uraniireducens* (+ inactivated G.u); the error bars represent the standard deviations of three replicate samples.

The BES for current generation was inoculated with *G. sulfurreducens* and subjected to batch mode via regular refreshing of the electrolytes over the long-term operation of 11 batches, and the metabolic activity of the anode biofilm was subsequently examined. As demonstrated, a decayed biofilm appeared with dead cells accumulated throughout the biofilm (Figs 1A and S1), and the current generation was impaired (Fig. 1D), as previously reported [21]. In contrast, at this point, when *Geobacter uraniireducens* was added, the decayed biofilm was rejuvenated, showing an active metabolism wholly (Figs 1B and S2). In addition, the current generation recovered (Fig. 1D), the redox activity improved (Fig. S3), and the electron transfer resistance decreased (Fig. S4), thus indicating enhanced electrochemical activity.

*Geobacter uraniireducens* is also an electroactive bacterium. It can form a biofilm on the anode and reduce the anode directly for current generation, similar to *G. sulfurreducens* [43]. However, it generates a much lower current than *G. sulfurreducens*. Unexpectedly, *G. uraniireducens* accounted for only a minor proportion (ca. 0.1%) of the biofilm (Fig. S5). These results indicated that the rejuvenated biofilm was derived from the recovery of the decayed *G. sulfurreducens* in the biofilm and *G. uraniireducens* contributed to neither the metabolic activity of the biofilm nor the recovery of current generation. We further evaluated the possibility that *G. uraniireducens* might be a nutrient or provide some substrates for *G. sulfurreducens* and then stimulate rejuvenation. The cell-free lysate of *G. uraniireducens* was added to the decayed BES. Neither the biofilm rejuvenated (Fig. 1C) nor the current generation recovered (Fig. 1D), suggesting that an active interaction between these two species is necessary for the rejuvenation of *G. sulfurreducens*.

### Prophage induction is inhibited after the addition of *G. uraniireducens*

**Figure 2:**
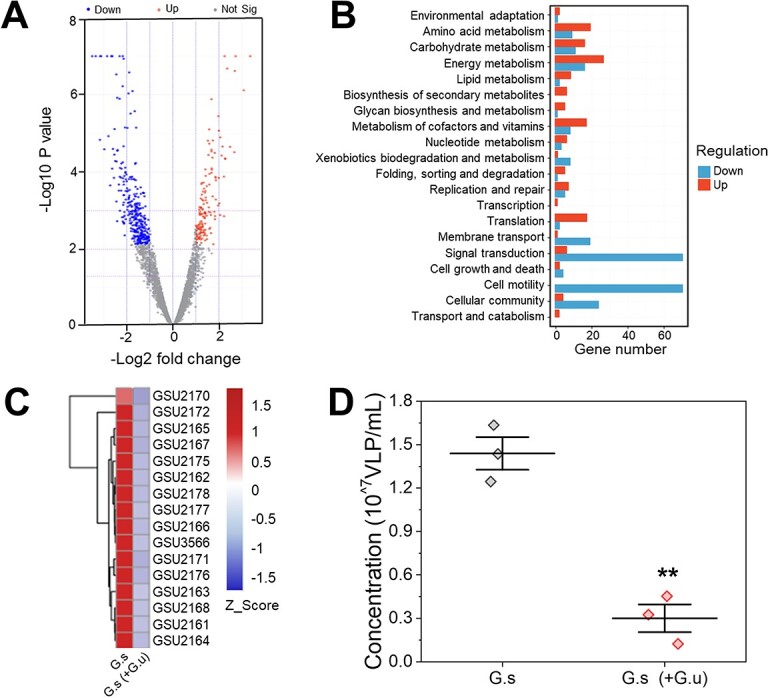
Transcriptomic profile and phage secretion in *G. sulfurreducens* biofilms after the addition of *G. uraniireducens*; (A) volcano plot illustrating the overall differences in gene expression between *G. sulfurreducens* biofilms treated with and without *G. uraniireducens*; genes with upregulated expression are represented by red dots, genes with downregulated expression are represented by blue dots, and genes not significantly differentially expressed (*P* > .05) are represented by gray dots; (B) the top 20 enriched KEGG pathways of differently expressed genes; (C) heatmap analysis of phage-related genes in prophage region 4, and red represents high gene expression abundance (Z_Score from 0 to 1.5), and blue represents low gene expression abundance (Z_Score from 0 to −1.5), and (D) the concentration of phage particles released in *G. sulfurreducens* biofilms; each data point represents one independent sample, and the horizontal and vertical bars stand for the mean of the data and the standard error of mean, respectively, and statistical analysis was performed by a two-tailed Student’s *t* test. ^*^^*^P < .005, *n* = 3.

To identify the possible interaction between *G. sulfurreducens* and *G. uraniireducens*, we further analyzed the transcriptome profiles of *G. sulfurreducens* EABs, and 492 genes of *G. sulfurreducens* were significantly affected in the presence of *G. uraniireducens* (Fig. 2A). Among them, 160 genes were upregulated, and 332 genes were downregulated. Specifically, two-thirds of the upregulated genes belonged to metabolic pathways (Fig. 2B), encompassing amino acid metabolism, carbohydrate metabolism, energy metabolism, lipid metabolism, and cofactor and vitamin metabolism. This result is consistent with the high viability of *G. sulfurreducens* after treatment with *G. uraniireducens* (Fig. 1B). In contrast, those phage-related genes were downregulated, particularly those genes in prophage region 4, which encodes *G. sulfurreducens* phage (Fig. 2C) [21], and the amount of phage particles secreted into the culture medium decreased (Fig. 2D) correspondingly. Thus, the presence of *G. uraniireducens* restrained the prophage induction of *G. sulfurreducens*. Prophage induction, particularly the activation of prophage region 4, has been shown to be the primary cause of *G. sulfurreducens* EAB decay [21]. The inhibited expression of phage-related genes must suspend the decay and then contribute to the rejuvenation of *G. sulfurreduces* biofilm.

Most of the rest prominently affected genes were related to stress-response systems, including upregulated genes related to genetic information processing (Fig. S6), such as genes encoding DNA and RNA polymerases, sigma factors [44], and cold shock-domain proteins (Csps) [45, 46], all of which are known to confer stress tolerance and protect cells by facilitating resistance to stress and repairing cellular damage; genes encoding OmpA proteins [47], HgtR-related proteins [48], and stress-responsive proteins [49], all of which could contribute to cellular adaptation to stresses by stabilizing the outer membrane; as well as downregulated genes mainly enriched in the functions of cell motility, signal transduction, cellular community, and membrane transport and are under the control of stress response systems. These results suggest that the presence of *G. uraniireducens* likely stresses *G. sulfurreducens*, thereby triggering its global stress response*.*

### Ecological competition causes the suppression of prophage induction

**Figure 3:**
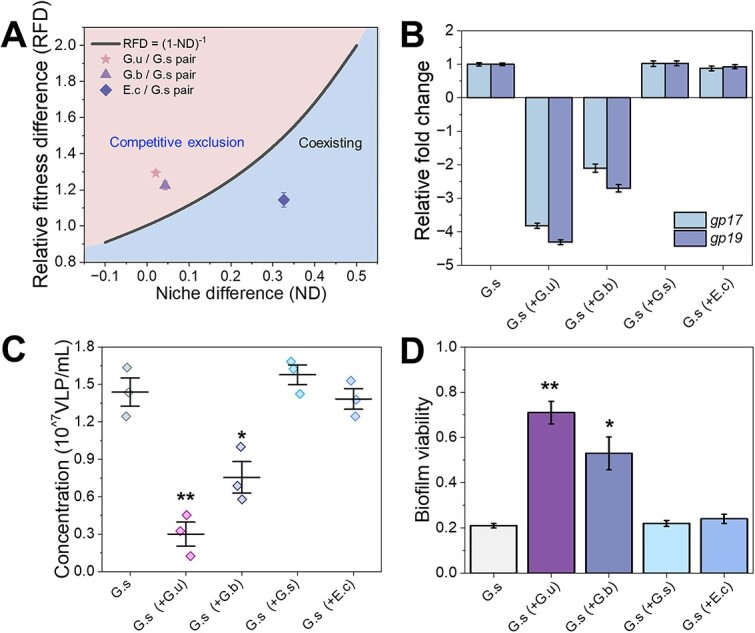
Ecological competition evaluation and prophage induction analyses; (A) the outcome of competition between *G. sulfurreducens* and three other species, *G. uraniireducens*, *G. bemidjiensis*, and *E. coli*; the curve RFD = (1-ND)^−1^ demarcates the boundary between competitive exclusion (light orange area) and coexisting (light gray area), and competition exclusion occurs when the calculated RFD is located in the light orange area (where RFD > (1-ND)^−1^), whereas coexisting occurs when RND is located in the light gray area (where RFD < (1-ND)^−1^), and (B) the relative fold change in the levels of the phage-related genes *gp17* and *gp19*; (C) the phage particle concentrations; the dots represent the separate individual data, whereas the horizontal and vertical bars stand for the mean of the data and the standard error of mean, respectively; (D) the total biofilm viability after the introduction of different bacterial species to *G. sulfurreducens* EAB, and statistical analysis was performed by a two-tailed Student’s *t-*test. ^*^P < .05, ^*^^*^P < .005, *n* = 3.

*Geobacter uraniireducens* and *G. sulfurreducens* are closely related electroactive *Geobacter* species that utilize similar electron donor and acceptor spectra. Thus, they should compete for the same resources when cocultured. We speculate that the addition of *G. uraniireducens* introduced competition stress to *G. sulfurreducens*. To test this hypothesis, we calculated the ND between *G. uraniireducens* and *G. sulfurreducens*. The ND measures the proportion of resources not shared by two species and reflects the level of niche overlap. In general, a smaller ND represents a greater overlap of niches, which indicates greater interspecies competition, and an ND value smaller than 0.1 usually indicates significant interspecies competition [40]. The results showed that the ND between *G. uraniireducens* and *G. sulfurreducens* was very minimal close to 0.02 (Fig. S7), corroborating the intense competition between *G. uraniireducens* and *G. sulfurreducens*. Furthermore, we measured the RFD, which represents the difference in competitive ability among species, and thereafter evaluated the competition outcome by comparing RFD with (1-ND)^−1^. According to modern coexistence theory, competitive exclusion occurs when RFD > (1-ND)^−1^; otherwise, coexistence dominates (RFD < (1-ND)^−1^) [50]. Between *G. uraniireducens* and *G. sulfurreducens*, the RFD was greater than that of (1-ND)^−1^ (Fig. 3A) indicating competitive exclusion. Thus, *G. sulfurreducens* must experience competition stress when *G. uraniireducens* is present. This result raises the question of whether the introduction of competition suppresses prophage induction in *G. sulfurreducens*.

In order to test the possibility that interspecies competition affects prophage induction, we tuned the intensity of competition by introducing two other bacterial species, *Geobacter bemidjiensis* and *E. coli*, with different NDs of 0.04 and 0.33, respectively, to the decayed *G. sulfurreducens* EAB. Thus, in theory, *G. bemidjiensis* and *E. coli* will exhibit high and low competition with *G. sulfurreducens*, respectively. In addition, *G. bemidjiensis* should undergo competitive exclusion from *G. sulfurreducens*, whereas *E. coli* should coexist with *G. sulfurreducens* (Fig. 3A). We also treated the decayed *G. sulfurreducens* EAB with metabolically active *G. sulfurreducens*, which theoretically should not introduce any competition. Similar to the *G. uraniireducens* treatment, the addition of *G. bemidjiensis* led to the downregulation of phage-related genes (Fig. 3B) and a decrease in phage secretion by *G. sulfurreducens* (Fig. 3C), indicating the suppression of prophage induction. Correspondingly, the biofilm viability (Fig. 3D) and current generation (Fig. S8) recovered significantly. In contrast, neither the addition of *E. coli* nor *G. sulfurreducens* treatment affected prophage induction (Fig. 3B, C) or had a significant impact on the biofilm viability (Fig. 3D) or current generation (Fig. S6) of *G. sulfurreducens*. Collectively, these results demonstrated that interspecies competition contributes to the suppression of prophage induction in *G. sulfurreducens*.

## Discussion

**Figure 4:**
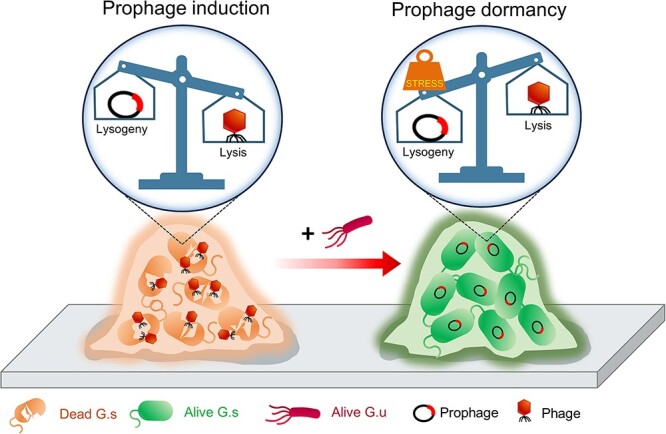
The model that introducing *G. uraniireducens* rejuvenates the decayed *G. sulfurreducens* EAB; prophage induction, a lysogenic to lytic switch, causes the decay of *G. sulfurreducens* EABs, and the addition of competitive species of *G. uraniireducens* exerts competition stress on *G. sulfurreducens*, which suppresses prophage induction to trigger the lytic to lysogenic transition in *G. sulfurreducens* and subsequently rejuvenates the decayed biofilm.

During the long-term operation of BESs, prophage induction in *G. sulfurreducens* causes the decay of *G. sulfurreducens* EAB and subsequently impairs current generation. Our study illustrated a therapy in which *G. uraniireducens* was introduced into the system to cure the phage attack and subsequently rejuvenated the decayed *G. sulfurreducens* EAB. Specifically, *G. uraniireducens* acted as a competitive species and probably applied competitive stress toward *G. sulfurreducens*, which triggered the suppression of prophage induction in *G. sulfurreducens* and thereafter allowed the metabolic activity of the *G. sulfurreducens* EAB to recover (Fig. 4). However, *G. uraniireducens* could not sustain proliferation in the system, possibly because it could not outcompete *G. sulfurreducens* for anode reduction, and during subsequent further operation, the abundance of the *G. uraniireducens* community declined gradually (account for less than 0.01%), and the *G. sulfurreducens* EAB started to decay again (Fig. S9). Therefore, it might be reasonable to regularly treat the *G. sulfurreducens* EAB with *G. uraniireducens* for maintaining long-term vitality. Even though *G. bemidjiensis* also exhibits competitive exclusion with *G. sulfurreducens*, it could not fully recover the biofilm vitality and current generation as *G. uraniireducens* (Figs 3D and S8). The reason should be that compared with *G. uraniireducens* and *G. sulfurreducens*, the competition between *G. bemidjiensis* and *G. sulfurreducens* is lower (Fig. 3A)*.*

Our results provide the evidence that interspecies competition can restrain prophage induction in bacterial cells. This finding is contrary to previous studies showing that interspecies competition generally stimulates lysogenized bacteria to evoke prophage induction, by which released phage particles kill or outcompete susceptible competitors [51, 52]. Even so, in either case, the competitive species acts as an external stressor on the cell. Previous studies have indicated that external stressors can trigger the host cell stress response to regulate the lytic-lysogenic switch of prophages [53–55]. Therefore, the different prophage decisions might be due to the different stress responses of the host cell. Even though, in either case, the host cell received competitive advantages, and in return, the phage achieved an increased level of fitness, suggesting symbiosis between phages and bacteria. Previous studies have also indicated that microbial stress response systems are the primary mechanism by which cells sense and respond to external stressors [56, 57]. Similarly, in our study, *G. sulfurreducens* activated the stress response system when competitive species were introduced, suggesting that the suppression of prophage induction in *G. sulfurreducens* was probably due to the inhibitory effect of the stress response system.

The finding that introducing competitive species could enhance the current generation of *G. sulfurreducens* sheds new light on the construction or design of synthetic microbial communities for BESs. In previous studies, the philosophy of “division of labor” guided the selection or engineering of functional bacterial species for constructing an efficient EAB [58]. Typically, one fermentative species is usually incorporated to in-charge of decomposing complex organics into small molecular organic acids, and another electroactive bacterium is selected to oxidize those organic acids for current generation. Thus, synergistic cooperation occurs, and interspecies competition has generally been considered to harm this process and therefore should be avoided. Importantly, our findings challenge this notion and reveal the advantage of “interspecies competition” in sustaining the performance of EABs. Actually, it has been supported by previous reports that mixed-species EABs usually perform better than pure culture biofilms [59], and coculturing competitive *Geobacter* species generated a greater current than single species [60]. Thus, our findings also provide new knowledge that can be used to help understand or explain the ecology of interspecies interactions in EABs.

Although mixed-species EABs are usually more sustainable than single *Geobacter* EABs, they always exhibit a much lower coulombic efficiency, which limits their application in BESs. In contrast, *Geobacter* species are the most efficient electroactive bacteria and generate the highest reported current; thus, these bacteria show the greatest promise in BESs. However, *Geobacter* EABs will decay during long-term operation. Previous studies have suggested applying external forces, such as ultrasound treatment and periodic polarization, to dislodge those decayed biofilms and thereafter to regrow fresh biofilms to make BESs sustainable [10, 61]. Apparently, these strategies have taken drastic measures and are sophisticated and energy-consuming. In addition, regrowing a new functional biofilm takes days. In contrast, our study provides a novel strategy involving treatment with competitive species to rejuvenate the decayed biofilm, which is time-saving (only requires several hours) and maneuverable. In particular, the competitive species could also be inoculated at the very beginning together with the working *Geobacter* species to sustain the longevity of the biofilm and thereafter enhance the performance of the BES (Fig. S10). Furthermore, considering the universality of prophages in bacterial species and their detrimental effects on biofilm decay, our study also has implications for defending against phage attack in other biofilm-based reactors.

## Conflicts of interest

The authors declare no conflict of interest.

## Funding

This research was supported by the National Science Fund for Excellent Young Scholars of China, grants no. 42222703, the National Natural Science Foundation of China, grant nos 42077218, 92251301, and the Project of Fujian Provincial Department of Science and Technology of China, grant no. 2022 J06015.

## Data availability

All data generated or analyzed during this study are included in this published article [and its supplementary information files].

## Supplementary Material

ISME_Supplementary_figures_wrae118
