# Supplementary material for: Interspecies ecological competition rejuvenates decayed Geobacter electroactive biofilm
Source: ISME J. 2024 Jun 25;18(1):wrae118. doi: 10.1093/ismejo/wrae118 (PMC11227281; doi:10.1093/ismejo/wrae118)
Supplement: ISME_Supplementary_figures_wrae118 [file isme_supplementary_figures_wrae118.pdf]

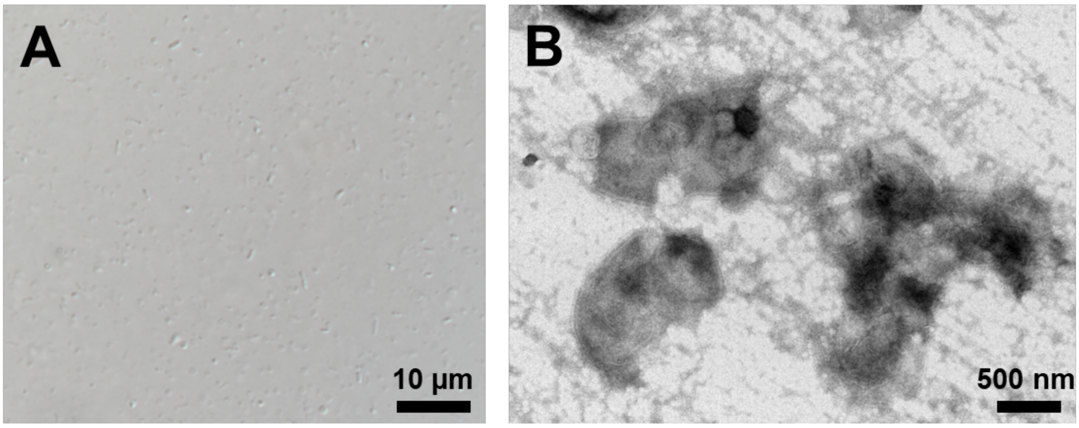

Figure S1. Microscopy images of *G. sulfurreducens* in decayed biofilm. (A) Phase contrast microscopy image. (B) Transmission electron micrograph.

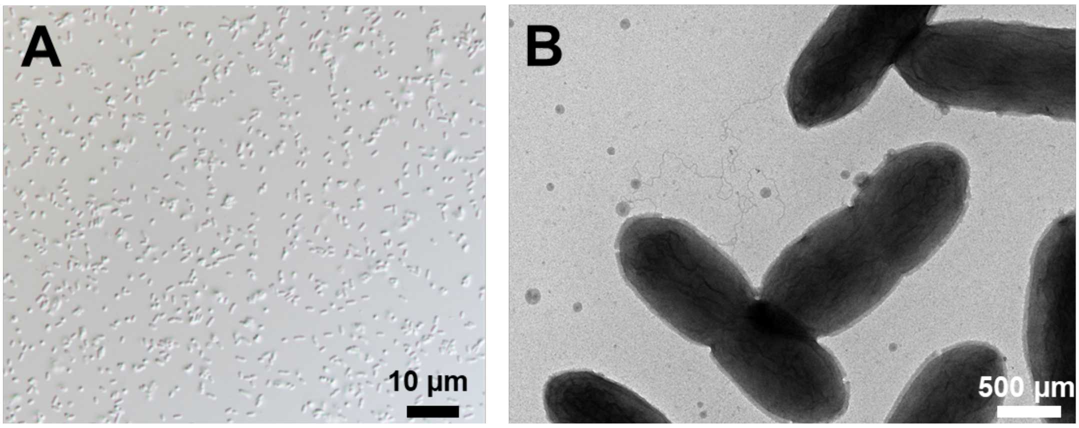

Figure S2. Microscopy images of *G. sulfurreducens* in rejuvenated biofilm. (A) Phase contrast microscopy image. (B) Transmission electron micrograph.

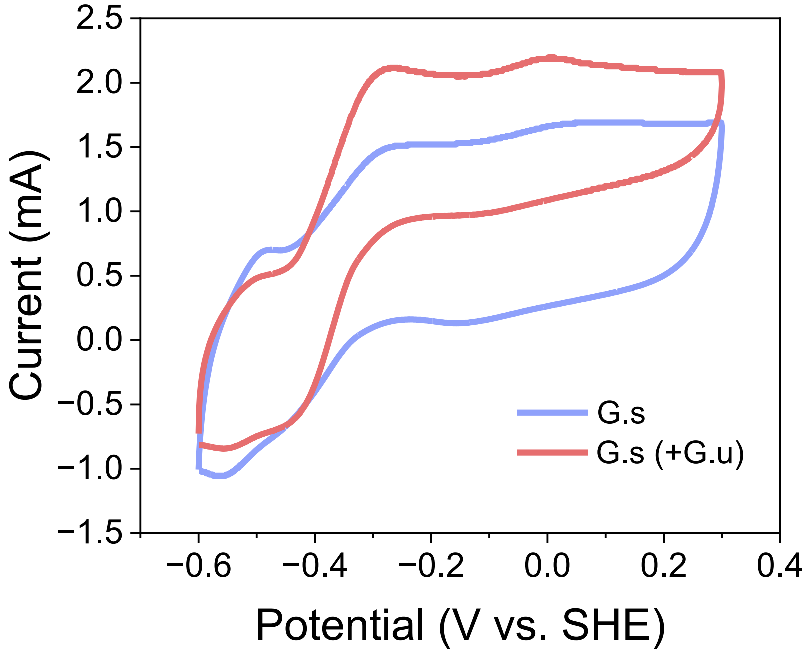

Figure S3. Cyclic voltammograms of *G. sulfurreducens* (G.s) electroactive biofilms with and without the addition of *G. uraniireducens* (G.u) under nonturnover conditions.

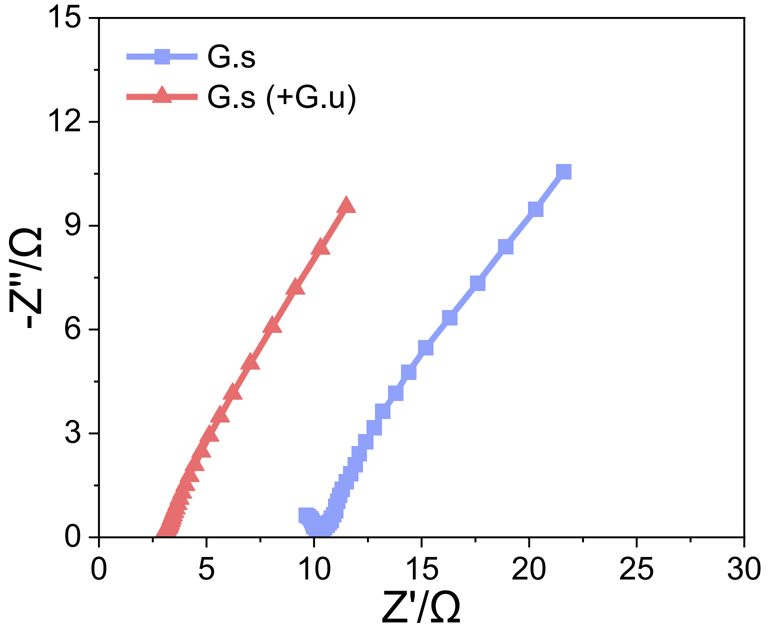

Figure S4. Electrochemical impedance spectra of *G. sulfurreducens* electroactive biofilms with and without the addition of *G. uraniireducens*.

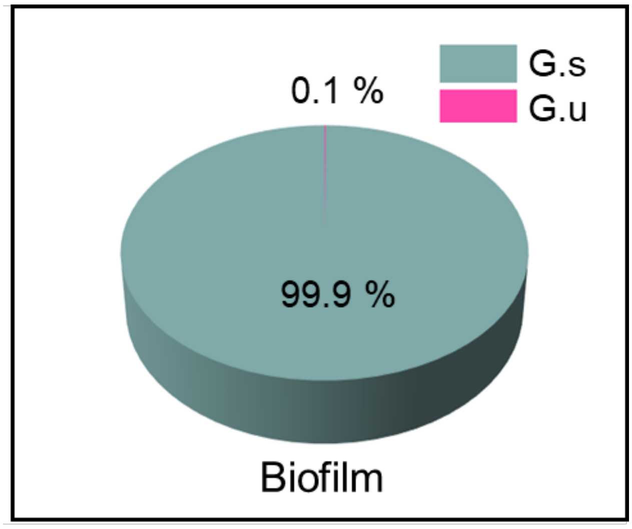

Figure S5. The relative abundances of *G. sulfurreducens* and *G. uraniireducens* in electroactive biofilms. The biofilm was collected at the end of the cycle and the amount of those two species were quantified by qPCR.

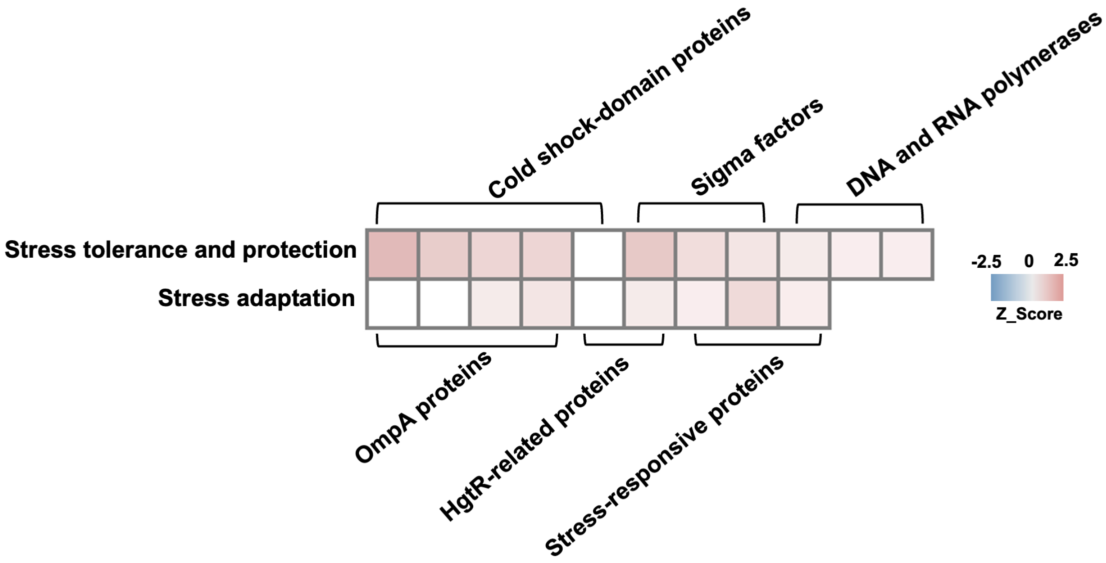

Figure S6. Expression of the genes assigned to the stress-response system of *G. sulfurreducens* after the addition of *G. uraniireducens*. Red represents high gene expression abundance (Z_Score from 0 to 2.5), and blue represents low gene expression abundance (Z_Score from 0 to -2.5).

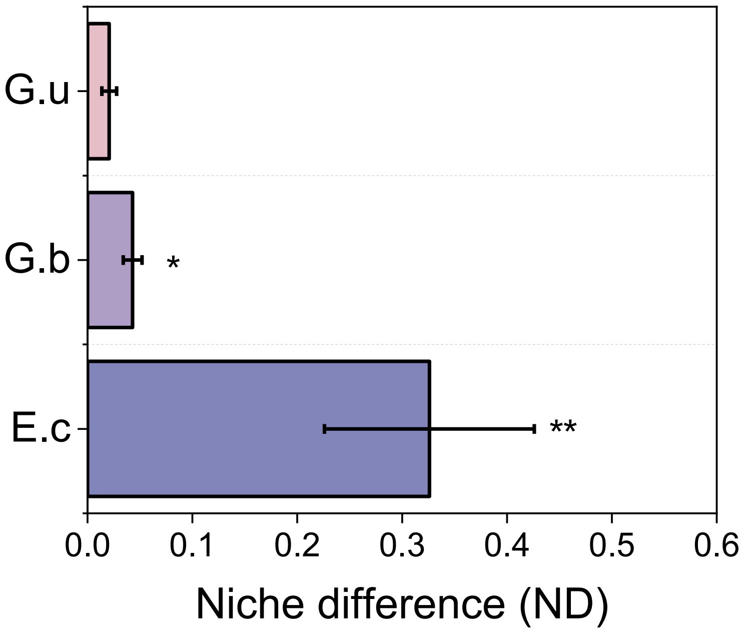

Figure S7. The niche differences between *G. sulfurreducens* (G.s) and *G. uraniireducens* (G.u), *G. sulfurreducens* and *G. bemidjiensis* (G.b), and *G. sulfurreducens* and *E. coli* (E.c). **P* < 0.05, ***P* < 0.005.

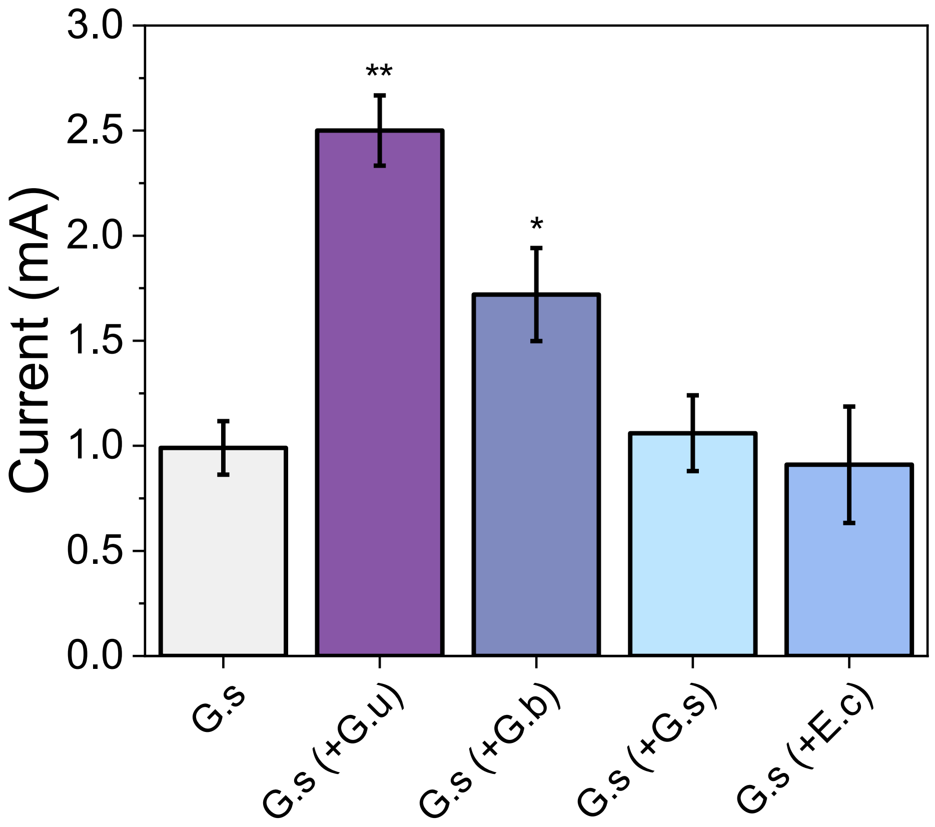

Figure S8. The maximal current generation of *G. sulfurreducens* electroactive biofilms after the addition of *G. uraniireducens* (G.u), *G. bemidjiensis* (G.b), *G. sulfurreducens* (G.s) or *E. coli* (E.c). *P < 0.05, *P < 0.005.

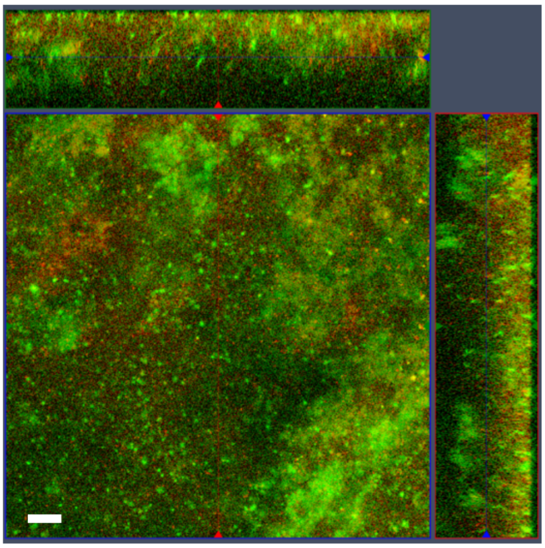

Figure S9. Confocal laser scanning microscopy image of rejuvenated *G. sulfurreducens* electroactive biofilm running for another two batch cycles after the *G. uraniireducens* treatment. The biofilm was stained with Live/Dead stain. Live cells were imaged as green, dead cells were imaged as red. There were dead cells accumulated in the biofilm. Scale bar represents 30 μm.

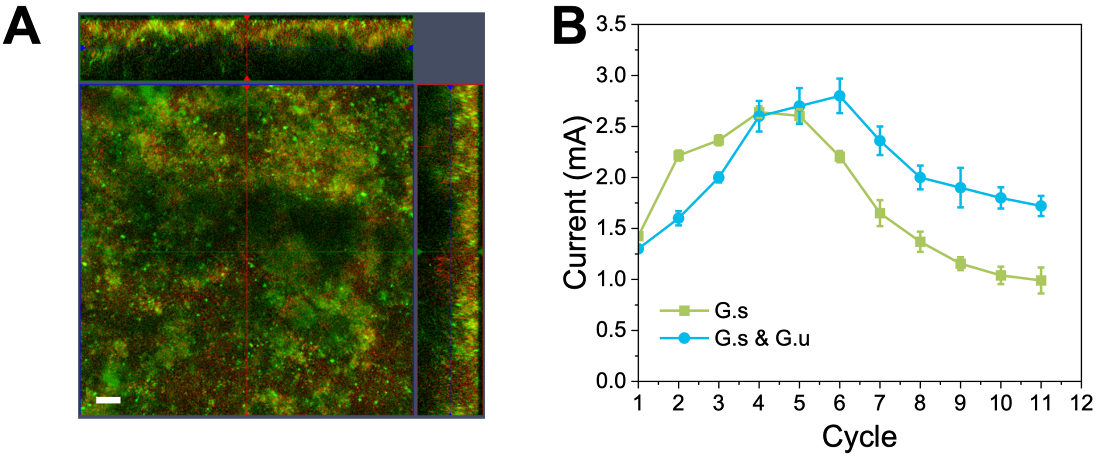

Figure S10. Characterization of *Geobacter* coculture electroactive biofilm. (A) Confocal laser scanning microscopy image of the *G. sulfurreducens* and *G. uraniireducens* coculture electroactive biofilm running through the 11th batch. These two species were cocultured at the beginning of the 1st batch. The biofilm was stained with Live/Dead stain. Live cells were imaged as green, dead cells were imaged as red. Scale bar represents 30 µm. (B) The current generation of *G. sulfurreducens* and *G. uraniireducens* coculture over 11 batches.

Table S1. The primers used for qPCR.

Primer name	Purpose	Sequence (5' to 3')
G.sF	qPCR for *GSU2597* gene of *G. sulfurreducens*	GAGGCCACTACGATTCTCCA
G.sR		AGGCTTCGATGAGAGTTCCG
G.uF	qPCR for *Gura_0266* gene of *G. uraniireducens*	GGCGACCTGGAGGATAAAG
G.uR		CCCAGCACCACTGTTCCTAT
G.bF	qPCR for *Gbem_3476* gene of *G. bemidiensis*	TGATACAAGCGGACCTGGTT
G.bR		GCAAAGAAAGCACTCGGGAT
E.cF	qPCR for *flgE* gene of *E. coli*	TGATACAAGCGGACCTGGTT
E.cR		TGATCCAAACAGCATTGCGA
gp19F	RT-qPCR for *gp19* gene of *G. sulfurreducens*	ACGTTCAGCACCGTGAAGT
gp19R		ATAGCTGCCGCCTTCCTTA
gp17F	RT-qPCR for *gp17* gene of *G. sulfurreducens*	CCCCTGACACTGAGGAAGAG
gp17R		TAAATACCGGTCCAAGCCTA
rpoDF	RT-qPCR for housekeeping gene *rpoD* of *G. sulfurreducens*	AGTTCTCGACGTACGCCACT
rpoDR		TCAGCTTGTTGATGGTCTCG
